# Comparison between minimally invasive, percutaneous osteosynthesis and locking plate osteosynthesis in 3-and 4-part proximal humerus fractures

**DOI:** 10.1186/s12891-015-0770-4

**Published:** 2015-10-14

**Authors:** Reinhold Ortmaier, Verena Filzmaier, Wolfgang Hitzl, Robert Bogner, Thomas Neubauer, Herbert Resch, Alexander Auffarth

**Affiliations:** Department of Traumatology and Sports Injuries, Paracelsus Medical University, Müllner Hauptstraße 48, A-5020 Salzburg, Austria; Department of Traumatology, Diakonissenkrankenhaus Schladming, Salzburgerstraße 777, A-8970 Schladming, Austria; Department of Biostatistics, Paracelsus Medical University, Müllner Hauptstraße 48, A-5020 Salzburg, Austria; Landeskrankenhaus Horn and Paracelsus Medical University, Spitalgasse 10, A-3580 Horn Salzburg, Austria

**Keywords:** Proximal humeral fractures, Locking plate, Percutaneous pin fixation, Humerusblock

## Abstract

**Background:**

The ideal method for the surgical treatment of proximal humeral fractures has not yet been found. We therefore conducted a retrospective matched-pair analysis and compared osteosynthesis with open reduction and internal fixation and that with an angular stable plate with minimally invasive, closed reduction, percutaneous fixation with the Humerusblock.

**Methods:**

During a study period of 3 years, we matched 30 patients treated with angular stable plates (group 1) for age, gender, fracture type and handedness (dominant or nondominant) to 30 patients treated using the Humerusblock (group 2). At a minimal follow-up of 24 months, clinical evaluation included the Constant-Murley score, the UCLA score and the Simple Shoulder Test. Subjective pain was evaluated using the VAS pain scale. Patients were asked to rate their subjective satisfaction of final outcome as excellent, good, satisfied or dissatisfied.

**Results:**

The mean CMS, UCLA score and SST differed significantly between groups 1 and 2 (60.9 vs 71.9, *p* < 0.01), (25.1 vs 29.5, *p* < 0.01) and (8.1 vs 9.4, *p* < 0.05), respectively. The VAS pain score was significantly lower in group 2 than in group 1 (1.2 vs 2.4; *p* < 0.01).

The mean abduction (109.7° vs 133.7°; *p* < 0.01) and anterior flexion (128.3° vs 145.7°; *p* < 0.01) were significantly worse in group 1. The mean operation time was significantly shorter in group 2 (117.3 vs 72.1, *p* < 0.01). Complications occurred in 30 % (group 1) and 23 % (group 2) of patients.

**Conclusions:**

In this study, the functional outcome is superior in the Humerusblock group. However, the general outcome after surgical treatment of 3-and 4-part fractures is moderate, and the complication rate has to be considered, even though it can be lowered with the use of minimally invasive implants.

## Background

Proximal humeral fractures (PHF) make up 5 % of all fractures [1]. Due to demographic changes in western countries, not only will the absolute number of PHF increase [2, 3] but also the number of complex PHF, due to an increased number of older patients with osteoporotic fractures [4]. Displaced and comminuted 3-and 4-part fractures are challenging for the surgeon, and no clear consensus for the best treatment strategy for each patient exists. Operative treatment comprises osteosynthesis and arthroplasty. Options for osteosynthesis range from locking plate fixation to intramedullary nailing, percutaneous pin fixation or the use of the Humerusblock [1, 5–8]. Currently, the workhorse for displaced 3-and 4-part proximal humeral fractures is open reduction and internal fixation using locking plate osteosynthesis. Biomechanical and clinical studies have shown high stabilities and union rates after locking plate procedures [6, 9, 10].

However, unsatisfactory complication rates of up to 40 %, after locking plate osteosynthesis show that the ideal joint-preserving method for treating proximal humeral fractures has not yet been found [11]. The Humerusblock is a k-wire based implant consisting of two locked, crossed k-wires, which allow for the minimally invasive, closed reduction and internal fixation of proximal humeral fractures. Although previous studies have shown that the Humerusblock provides all of the advantages of a minimally invasive device, high rates of pin perforation and high implant removal rates have been observed [8, 12–15].

In this retrospective, matched-pair analysis, we set out to compare the clinical and radiological results of open reduction and internal fixation using angular stable plating, with those of minimally invasive, semi-rigid, Humerusblock osteosynthesis.

## Patients and methods

The study was reviewed and approved by the hospital institutional review board (Ethikkommission für das Bundesland Salzburg). Written informed consent was obtained from all the patients for the publication of this study. Consent to publish personal details in Tables 2, 3 and 4 was obtained from all participants.

**Fig. 1:**
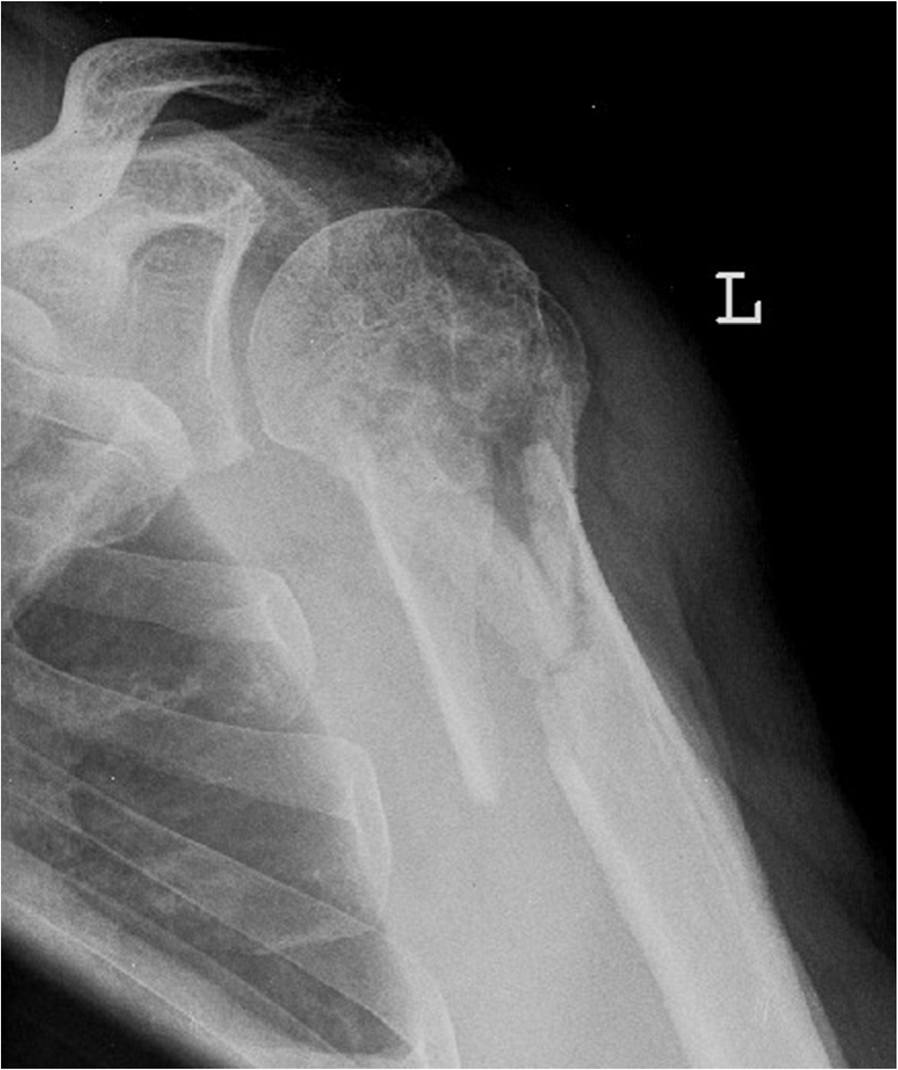
Limitation of the Humerusblock. The long metaphyseal fracture line reaching far below makes it impossible to stabilize the fracture with the Humerusblock

From 2009 to 2012, 291 patients underwent osteosynthesis for proximal humeral fractures using either the PHILOS Plate (Synthes, Oberdorf, Switzerland) or the Humerusblock (Synthes, Oberdorf, Switzerland). 139 patients were treated with PHILOS plate, and 152 were treated with the Humerusblock. In our institutions, the PHILOS plate and the Humerusblock are used. Usage of either implant depends on the preference of the surgeon. The Humerusblock can be used for subcapital and intra-articular humeral fractures (AO/OTA classification A3, B1-3, C1-3). Due to its design, the only limitation of the Humerusblock is the use in AO A2 metaphyseal fractures with fractures level extending far below the surgical neck (Fig. 1). Use of either the PHILOS plate or the Humerusblock was decided by the surgeons with the patient and was not randomized or blinded. Demographic data were used to retrospectively review a prospectively gathered database of the 291 patients treated with either the PHILOS plate or the Humerusblock to perform a matched-pair analysis. Patients were matched for age (within +/−3 years), gender, handedness (dominant or non-dominant), affected side and fracture type (3-or 4-part proximal humeral fracture by Neer’s classification system) [16]. The matching procedure was blinded to the outcome. The minimum follow-up was 24 months after surgery. Exclusion criteria were head-split fractures, lesions of the brachial plexus, pathologic fractures, dementia, previous surgery on the affected shoulder, heavy tobacco abuse, alcohol abuse or steroid intake.

86 Patients (43/group) fulfilled the matching criteria and were contacted to return for clinical and radiological evaluation. Of those, 15 died and 11 did not return for evaluation.

Therefore, 30 patients treated using the PHILOS plate were matched to 30 Humerusblock patients according to the matching criteria. In both groups, there were 20, 4-part fractures and 10, 3-part fractures.

**Fig. 2:**
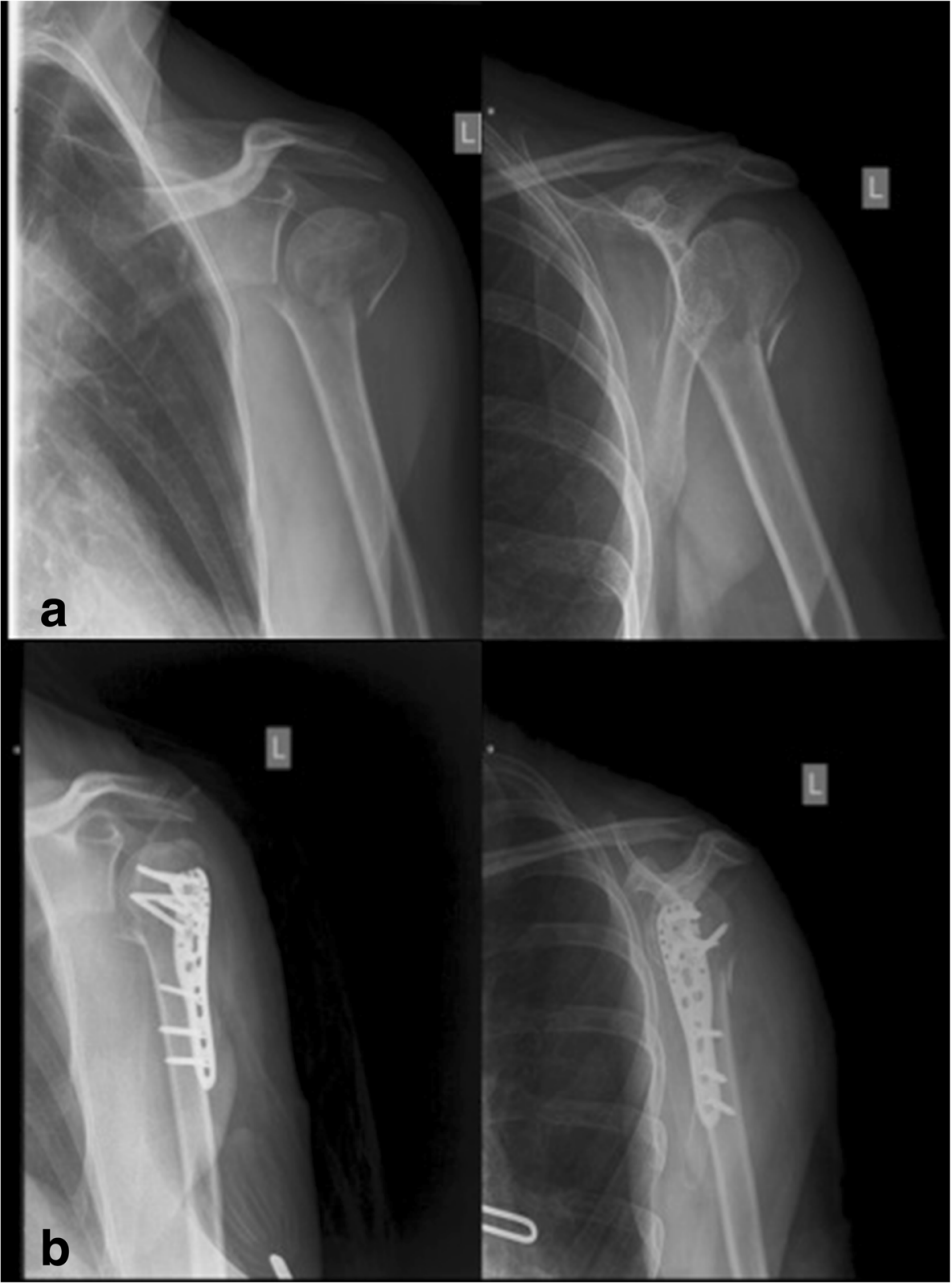
A valgus, impacted 3-part fracture of a left shoulder treated using the PHILOS plate **a**. X-rays in 2 planes postoperatively **b**. Matching criteria and outcome are illustrated in Table 2

**Fig. 3:**
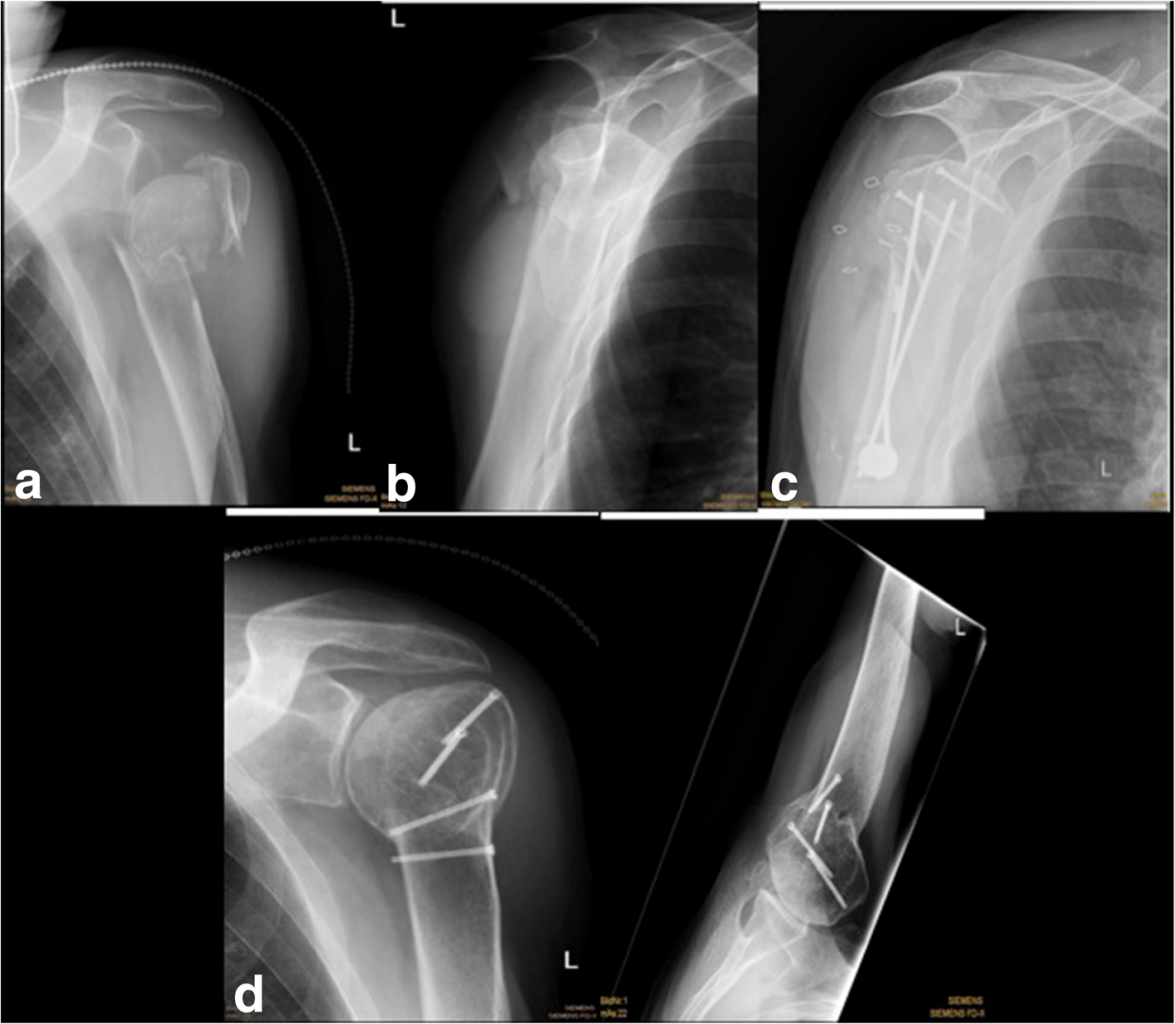
A valgus, impacted 3-part fracture of a left shoulder treated using the Humerusblock **a**, **b**. X-ray postsurgical after Humerusblock **c**. X-rays in 2 planes at final follow-up after 40 months **d**. Matching criteria and outcome are illustrated in Table 2

Group 1 (PHILOS) comprised 30 patients (17 female, 13 men) with a mean age of 61.3 years (range, 36–80 years). Group 2 (HB) also comprised 30 (17 female, 13 men) patients with a mean age of 61.7 years (range, 37–78 years). Table 1 shows details of the matched pairs. Figures 2 and 3 show an example of a matched-pair analysis, and Table 2 shows the demographics and outcomes of the example.

**Table 1:** Patient demographics

	Group1	Group2	Overall
Age (range)	61.3 (36–80)	61.7 (37–78)	61.5 (36–80)
Gender			
Male	13	13	26
Female	17	17	34
Handedness			
right	23	23	26
left	7	7	14
Side (injured arm)			
right	18	18	36
left	12	12	24
OR time min (range)	117.3 (77–208)	72.1 (31–206)	94.7 (31–208)
Accident			
Low energy (home fall, pedestrian)	23	17	40
Traffic (bike, motorcycle, car)	7	13	20
ASA			
1	8	10	18
2	17	13	30
3	5	7	12
Fracture type			
4 part	20	20	40
3 part	10	10	20
valgus	17	17	34
varus	13	13	26

**Table 2:** An example of two matched patients

	patient group 1	patient group 2
gender	female	female
age	62	64
handedness	left	left
affected arm	left	left
fracture type	Valgus impacted 3-part	Valgus impacted 3-part
accident	bike	Home fall
ASA	2	2
operation time	102 min	71 min
CMS	60	80
UCLA	22	26
SST	6	8
VAS	3	1

X-rays of the matched patients are illustrated in Fig. 1 and 2

### Surgical technique

All of the procedures on patients in both groups were performed in the beach chair position. General anesthesia in combination with an interscalene block were used in all procedures, and all patients received perioperative intravenous antibiotic prophylaxis. In group 1, (PHILOS) a deltopectoral approach was used in all of the patients. First, the tuberosities were tagged with non-absorbable number 2 or 3 sutures behind the rotator cuff insertion. Then, the humeral head was reduced and fixed with k-wires. The PHILOS plate was then adapted, and the tuberosities were reduced by tying them together over the plate. The PHILOS plate was temporarily secured at the humeral shaft with k-wires, and a non-locking screw was introduced through the plate in the shaft. Holes for the head screw were made by subchondral drilling under fluoroscopy control in order to avoid perforating the joint. Before the end of surgery, the final result was assessed by fluoroscopy.

**Fig. 4:**
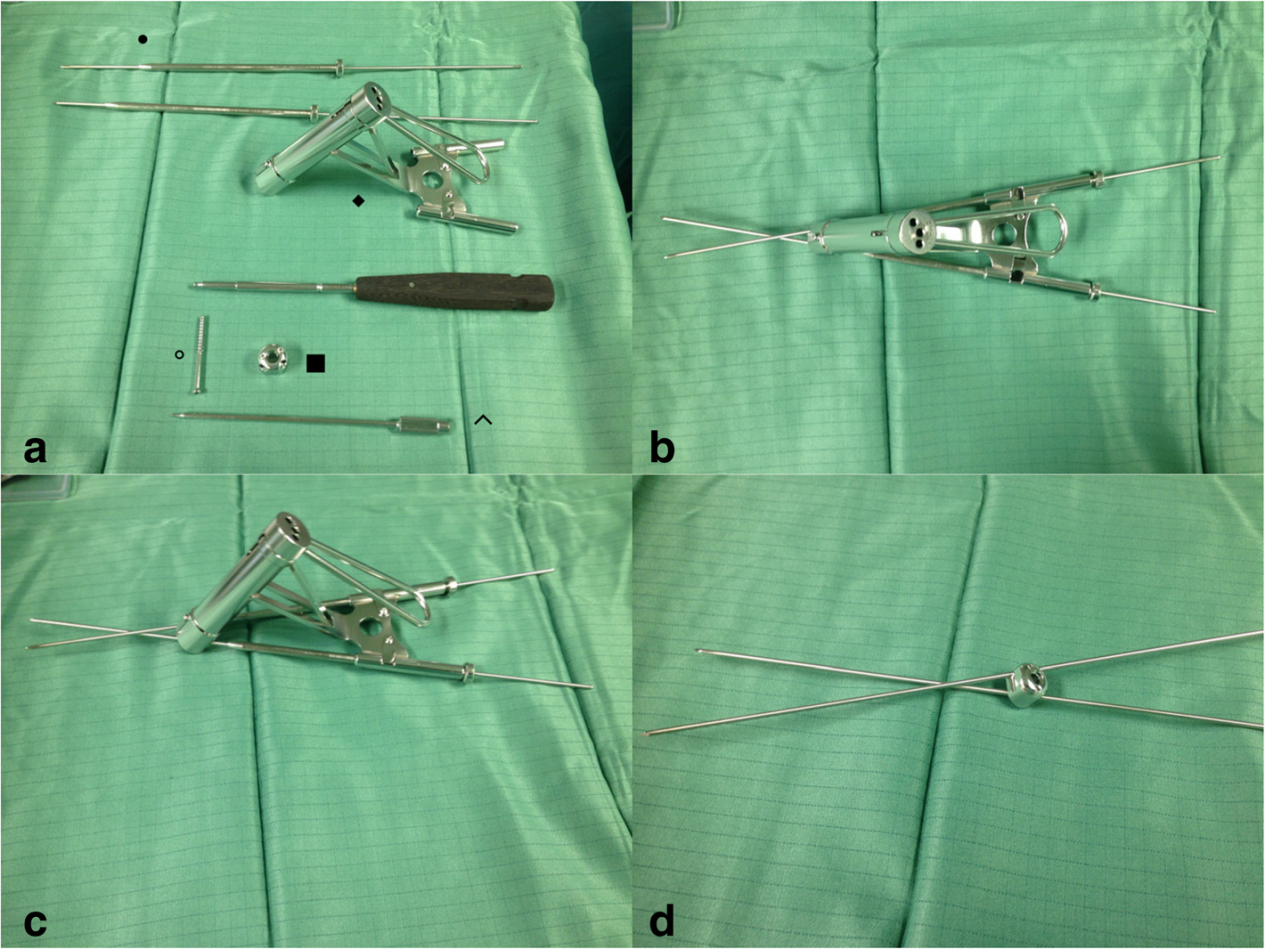
The Humerusblock implant and instruments.**a** shows ● Kirschner-wire centering sleeve with two 2.5 mm Kirschner wires. ◆ Insertion guide and drill sleeve for Humerusblock. ■ Humerusblock with two offset canals for the Kirschner wires and two headless pins for locking the Kirschner wires in the Humerusblock. ° 3.5 mm self-tapping cortex screw to fix the Humerusblock to the lateral aspect of the humeral bone. ^Special screw driver for Humerusblock to fix the headless pins.**b** shows Humerusblock insertion guide together with Kirschner-wire centering sleeve and two 2.5 mm Kirschner wires. **c** shows Humerusblock insertion guide, Kirschner-wire centering sleeve and Kirschner wires from lateral. **d** Kirschner wires crossing over at an angle of 25° through the Humerusblock

The Humerusblock is made of stainless steel and is made for the fixation of 2 k-wires up to 2.5 mm in diameter (Fig. 4). The 2 lateral canals for the k-wires are at an angle of 35° at the lower plane of the implant and at a 25° angle to each other, which makes the k-wires cross over and diverge in the humeral head. The k-wires are locked in the Humerusblock by small pins. The Humerusblock itself is secured at the shaft by a 3.5 mm self-tapping screw at the shaft.

**Fig. 5:**
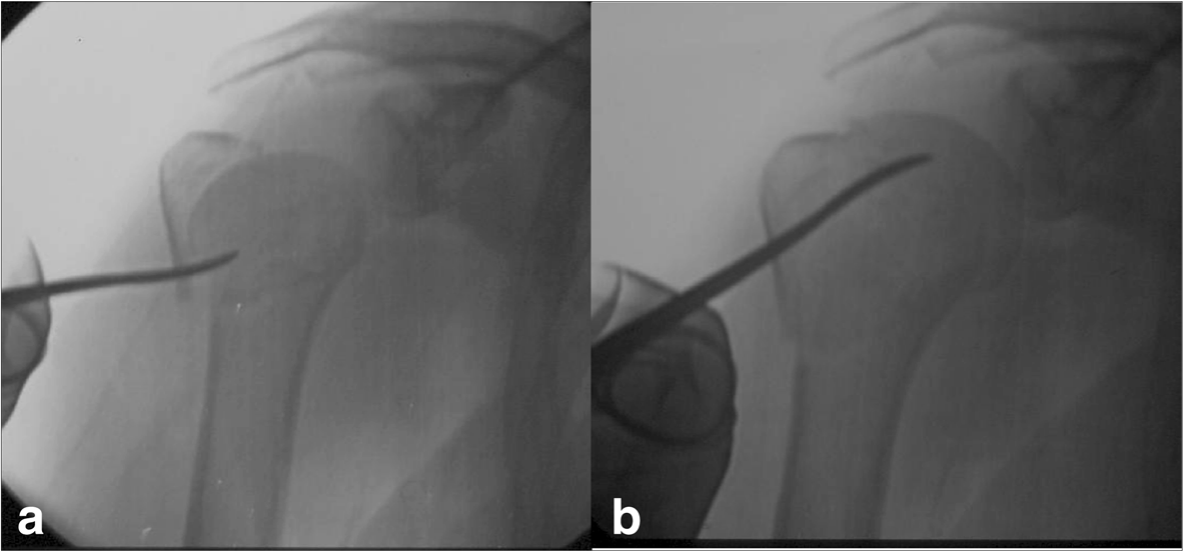
An elevator is introduced into the fracture gap *via* a small skin incision to reduce the head by lifting it **a**, **b**

The Humerusblock is inserted *via* a 3 cm skin incision at the lateral aspect of the upper arm, approximately 5 cm distal to the subcapital fracture level. The Humerusblock is fixed to the shaft with a self-tapping cannulated 3.5 mm screw. Two 2.5 mm k-wires are introduced into the shaft up to the fracture level in the so-called “waiting position”. Then, the closed reduction under manual traction and fluoroscopy control is performed. The surgeon holds the arm and reduces the fracture, and the assistant drills the k-wires to the subchondral level. The reduction of the tuberosities and/or the lifting or derotating of the head is performed *via* small skin incision using hooks and elevators, as seen in Fig. 5. Tuberosities are percutaneously fixed using 2.7 mm or 3.0 mm cannulated screws. Finally, the two 2.5 mm k-wires are tightened and a few millimeters are cut off of the Humerusblock.

### Postoperative rehabilitation

Patients in group 1 were treated with immobilization of the shoulder in a sling for 2 weeks. Active finger, wrist and elbow movement was allowed on the first postoperative day, and pendulum exercises of the shoulder were started at least 14 days postoperatively. Active abduction up to 90° was started after the sling was removed, depending on surgeon-specified guidelines. Heavy manual work and resistive exercises were allowed 8 to 12 weeks postoperatively.

The postoperative rehabilitation protocol of patients in group 2 included wearing a shoulder sling for 4 weeks. Finger and elbow movements were allowed immediately postoperatively. Active abduction and anterior elevation were allowed after sling removal, while load-bearing and heavy manual work was allowed after 10–12 weeks.

### Data collection/clinical and radiological evaluation

The Constant-Murley score (CMS) [17], the UCLA score [18] and the Simple Shoulder Test (SST) [19] were determined. The Constant-Murley score includes the pain score, functional assessment, range of motion and strength measurement with a maximum score of 100 points. The UCLA score includes pain, function, satisfaction and strength with a maximum score of 35 points. The Simple Shoulder Test comprises 12 yes-or no-response questions to objective and subjective items. The visual analog pain scale (VAS) [20] was used to rate the patient’s subjective pain. Range of motion was measured using a goniometer. The patients were also asked to rate their subjective satisfaction at the final follow-up as either excellent, good, satisfied or dissatisfied. The clinical evaluation was performed by one independent examiner (OR) who was not part of the surgical team.

Radiological evaluation included an X-ray of the affected shoulder in at least 2 standard projections anteroposterior (AP) and axial and/or Y views. The X-ray evaluation was focused on malreduction, malunion, nonunion, AVN, loss of reduction and screw or pin perforation.

### Statistical analysis

Data consistency was checked and data were screened for outliers and normality by using quantile plots. Paired Student’s t-tests were used to compare variables between groups. All reported tests were two-sided, and *p*-values < 0.05 were considered to be statistically significant. All statistical analyses in this report were performed with STATISTICA 10 (Hill, T. & Lewicki, P. Statistics: Methods and Applications. StatSoft, Tulsa, OK).

## Results

### Group 1 PHILOS plate

After a mean follow-up of 38.4 months (range, 26–56 months), patients scored 60.9 points (range, 15–87) on the CMS. The mean UCLA score was 25.1 points (range, 15–35), and the mean SST was 8.1 points (range, 1–12), respectively. The mean abduction was 109.7° (range, 40–170), and mean anterior flexion was 128.3° (range, 40–170). The mean VAS pain score was 2.4 (range, 0–7). The mean operation time was 117.3 min (range, 77–208).

At final follow-up, 7 patients rated their outcome as excellent, 5 as good, 10 as satisfied and 8 as dissatisfied.

Complications (Table 3) occurred in 30 % of patients (9 patients). In 5 patients, (17 %) secondary screw cut-out due to varus collapse was seen; of those, 5 patients were treated with reosteosynthesis at 6, 8, 10, 11 and 12 weeks after initial surgery.

**Table 3:** Complications and outcome after treatment using the PHILOS plate

Patient	Gender	Age	Fracture type	ASA	Type	Reoperation (weeks after fracture treatment)	CMS	UCLA	SST	VAS	Satisfaction
1	male	75	4 part	2	Screw cut out	6	54	25	7	4	dissatisfied
2	female	70	4 part	2	Screw cut out	8	50	18	7	1	satisfied
3	male	62	4 part	3	Screw cut out	10	45	22	6	3	dissatisfied
4	female	71	3 part	2	Screw cut out	11	51	29	7	1	satisfied
5	female	74	4 part	2	Screw cut out	12	50	25	8	3	dissatisfied
6	female	78	4 part	3	AVN		44	24	7	7	dissatisfied
7	female	67	3 part	3	AVN		15	13	1	7	dissatisfied
8	female	80	4 part	2	AVN		30	15	5	3	dissatisfied
9	female	75	4 part	3	AVN		37	15	4	3	dissatisfied

Four patients (13 %) showed signs of avascular necrosis of the head. Presently, the 4 patients refuse to undergo shoulder arthroplasty. Implant removal was performed in 12 (40 %) of the patients after bony healing.

### Group 2 humerusblock

The mean follow-up of patients in group 2 was 36.1 months (range, 25–50 months). The mean CMS score was 71.9 points (range, 34–88). The mean UCLA score was 29.5 points (range, 17–34), and the mean SST was 9.4 points (range, 4–12), respectively. The mean abduction was 133.7° (range, 50–170), and mean anterior flexion was 145.7° (range, 60–180). The mean VAS pain score was 1.2 (range, 0–6). The mean operation time was 72.1 min (range, 31–206).

At final follow-up, 10 patients rated their outcome as excellent, 6 as good, 10 as satisfied and 4 as dissatisfied.

Postoperative complications (Table 4) occurred in 7 patients (23 %). Three patients (10 %) had a loss of reduction. Reosteosynthesis was performed 1, 2 and 3 weeks postoperatively. Of those who received reosteosynthesis, 1 patient was dissatisfied, and the other two were satisfied at the final follow-up. The dissatisfied patient had a CMS score of 54 points, and the satisfied patients had CMS scores of 60 and 64 points. Two patients (7 %) showed signs of AVN at the final follow-up. Presently, neither of these two patients wants additional surgery. 2 (7 %) patients underwent retrieval of the pins due to pin perforation with the pin tips aimed at the glenoid surface.

**Table 4:** Complications and outcomes after treatment using the Humerusblock

Patient	Gender	Age	Fracture type	ASA	Type	Reoperation (weeks after fracture treatment)	CMS	UCLA	SST	VAS	Satisfaction
1	female	70	4 part	2	loss of reduction	1	54	21	8	4	dissatisfied
2	female	54	4 part	2	loss of reduction	2	60	27	9	2	satisfied
3	male	62	4 part	3	loss of reduction	3	64	28	9	2	satisfied
4	female	48	3 part	2	pin perforation	2	70	30	10	0	satisfied
5	male	67	4 part	3	pin perforation	3	60	21	8	4	dissatisfied
6	female	72	4 part	2	AVN		48	18	8	5	dissatisfied
7	female	78	4 part	2	AVN		34	17	4	6	dissatisfied

None of the patients with pin perforation experienced delayed healing or were dissatisfied at the final follow-up. Implant removal was performed in 16 (42 %) of the patients after bony healing.

### Comparison of the groups

The mean functional outcome scores (CMS, UCLA and SST) differed significantly between groups 1 and 2: (60.9 vs 71.9, *p* < 0.01), (25.1 vs 29.5, *p* < 0.01) and (8.1 vs 9.4, *p* < 0.05), respectively. The mean abduction (109.7° vs 133.7°; *p* < 0.01) and anterior flexion (128.3° vs 145.7°; *p* < 0.01) was significantly worse in group 1.

The VAS pain score was significantly lower in group 2 compared to group 1 (1.2 vs 2.4; *p* < 0.01). The mean operation time differed significantly between the groups (117.3 vs 72.1, *p* < 0.01).

## Discussion

Currently, angular stable plating is widely used in osteosynthesis for the treatment of complex 3-and 4-part fractures of the proximal humerus [10]. Despite many reports showing considerably improved results after angular stable plating, complication rates higher than 40 % are reported [10]. This give rise to the idea that the ideal method for treatment of 3-and 4-part PHFs has not been found. In this study, we found the functional results and postoperative pain according CMS, UCLA score, SST and visual analogue pain scale were significantly better after the use of the Humerusblock than after that of the PHILOS angular stable plate. According to a systemic review analyzing results after angular stable plating, a mean CMS score of 72 points for 3-part fractures and 66 points for 4-part fractures were reported [10]. Those results are slightly better than our results of a mean CMS score of 60.9 points.

Range of motion seems to be very important especially for older people in order to manage their activities of daily living. For this reason, in older patients, perhaps it is more important to restore range of motion than strength. Surprisingly, only a few studies specifically report on range of motion after plating [10]. However, in this study, abduction and anterior flexion were found to be significantly better in the Humerusblock group than in the PHILOS plate group.

There are only a few reports of results after the use of the Humerusblock [8, 12, 13]. Brunner *et al*. [13] analyzed in a prospective case series 58 patients with a mean CMS of 73.6 points, abduction of 107° and anterior flexion of 119.2°. Bogner *et al*. [8] found in 51 3-and 4-part fractures after a mean follow up of 33.8 months, a mean CMS of 61.2 points for 3-part and 49.5 points in 4-part fractures in elderly patients, with a mean overall CMS of 71.6 points and abduction and anterior flexion of 133° and 143.5°. Therefore, our results are in line or even slightly better than the results reported in the literature.

Complication rates after angular stable plating up to 49 % [10] and reoperation rates up to 44 % [21] are reported. The most common complications are screw perforations with rates between 8 %–20 %, avascular necrosis between 10 %–33 %, loss of fixation up to 16 %, impingement up to 6 %–11 % and infection between 4 %–19 % [10, 21]. In this study, we found complication and reoperation rates of 30 % for patients treated with the PHILOS plate and 23 % for the Humerusblock. Radiologic signs of AVN were found in 4 patients in the PHILOS group and in 2 in the Humerusblock group. Occurrence of AVN is influenced not only by surgical factors but also by nonsurgical factors, such as fracture type, medial hinge, short calcar fragment or head split fracture, as well as the comorbidities of the patient [22]. However, nonanatomical reduction and extended soft tissue dissection are suggested to promote AVN development [22, 23]. The Humerusblock is removed from the fracture zone without harming soft tissue in the injured area. Reduction is performed in a closed or percutaneous manner, thus preserving remaining periosteal bridges between fracture fragments and reducing the risk of AVN [8, 12, 13]. Unlike pin perforation after treatment using the Humerusblock, screw cut-out after angular stable plating is a serious complication [11]. In the literature, screw perforations seem to be one of the most frequent complications after plating, with reported rates up to 20 % [11, 24]. In a study by Jost *et al*. [11], 57 % of patients with screw perforations showed glenoid destruction, and this represents a devastating complication. In our study, 5 patients with screw perforations needed reosteosynthesis. In contrast to screw perforation, in angular stable plating, pin perforation after treatment using the Humerusblock, as long as the tips of the k-wires do not aim at the glenoid surface, is considered a minor complication. Quite to the contrary, the Humerusblock allows for the dynamic stabilization and controlled sintering and fracture consolidation of the head fragment. In this study, pin perforation occurred in 2 patients (7 %), but rates up to 41 % are reported in the literature [14]. In the studies by Brunner *et al*. [13] and Bogner *et al*. [8], secondary impaction of the head leads to k-wire perforation in 22 % and 10 % of patients, respectively. Pin perforation can be easily detected in standard x-rays. Treatment is simple and doesn’t influence final outcome or bony healing [13]. If pin perforation occurs before bony healing and the k-wires aim at the glenoid surface, retrieval at the subcortical level is performed under local anesthesia. If the k-wires don’t aim at the glenoid surface, immediate k-wire removal after bony healing and before sling removal is performed. In general, the Humerusblock is not required to be removed. As mentioned, such as in angular stable plates, only in cases of k-wire perforation or due to the wish of the patient should the Humerusblock be removed.

Loss of reduction is a well-known complication after osteosynthesis of complex 3-and 4-part fractures [10, 11, 21]. In this study, a loss of reduction was observed in 3 patients after treatment using the Humerusblock and in 5 patients after treatment using the PHILOS plate. In the Humerusblock group, all 3 patients were successfully treated with reosteosynthesis using the Humerusblock again, and 5 patients in the PHILOS group were successfully treated with reosteosynthesis using the PHILOS plate again. In angular stable plating, a loss of reduction often causes varus collapse, which leads to screw perforation. Osteoporotic bone angular stable plates may provide too much stiffness, which can lead to stress between the implant/bone interface leading to screw cut-out or micromotion under threshold for callus formation [25, 26]. However, excessively elastic implants lead to early failure. With the Humerusblock, k-wires are fixed to the block preventing migration. The semirigid design reduces peak stresses at the bone implant interface and allows the humeral head to sinter [25].

Operation time in the Humerusblock group was 45.2 minutes faster than in the PHILOS group. However, if the Humerusblock is removed after bony healing, a short, second operation is planned and performed.

This study has several limitations. Its small sample size and dropout rate of 30 % as well its retrospective design may limit its validity. However, the main focus in this study was to increase the comparability of both cohorts, which is based on an exact and detailed matching process. Although, many reports about treatment of PHF and angular stable plating exist, data heterogeneity concerning patient population, fracture type and outcome measures makes it difficult to draw definitive conclusions concerning treatment suggestions [10].

In this study, we found the use of the Humerusblock in the treatment of complex 3-and 4-part fractures was superior to angular stable plating in regard to postoperative outcome, patient satisfaction and complication rate. Therefore, for us, the Humerusblock is a good, if not superior, alternative to angular stable plates in the treatment of proximal humeral fractures. However, the outcome after surgical treatment of 3-and 4-part fractures is moderate and the complication rate is high.

## Conclusion

The general outcome after surgical treatment of 3-and 4-part fractures is moderate and the complication rate is high. However, in this study, the functional outcome is superior, the pain lower and the operation time is faster in the Humerusblock group. For us, the better outcome can be explained by the minimally invasiveness of the implant. The reduction is performed closed or *via* stab incision, which leaves periosteal bridges and the fracture hematoma intact. As every surgical procedure the Humerusblock has a certain learning curve and beginners are advised to start with simple fractures. Once learned, the Humerusblock is a fast, cheap and secure technique with represents a good alternative to the widely used angular stable plates in the treatment of proximal humeral fractures. However, prospective, randomized controlled, multicenter trials with high numbers of participants are necessary to draw definitive conclusions.

## Abbreviations

PHF: Proximal humeral fractures
AO/OTA: AO/OTA Fracture and Dislocation Classification
CMS: Constant-Murley score
UCLA score: University of California-Los Angeles Shoulder Score
SST: Simple Shoulder Test
VAS: The visual analog pain scale
AP: Anterior Posterior
AVN: Avascular necrosis
OR: Operation time
ASA: Anesthesia Physical Classification System
